# Development of a Caffeine Content Table for Foods, Drinks, Medications and Supplements Typically Consumed by the Brazilian Population

**DOI:** 10.3390/nu14204417

**Published:** 2022-10-21

**Authors:** Pedro Lucas de Amorim Rocha, Anna Luisa Caldeira Lima, Bryan Saunders, Caio Eduardo Gonçalves Reis

**Affiliations:** 1 Human Nutrition Graduate Program, School of Health Science, University of Brasília, Brasília 70910-900, Brazil; 2 Department of Nutrition, School of Health Science, University of Brasília, Brasília 70910-900, Brazil; 3 Applied Physiology and Nutrition Research Group, School of Physical Education and Sport, Rheumatology Division, Faculdade de Medicina FMUSP, University of São Paulo (USP), São Paulo 01246-903, Brazil; 4 Laboratory of Nutritional Biochemistry, Department of Nutrition, School of Health Science, University of Brasília, Brasília 70910-900, Brazil

**Keywords:** caffeine, table, nutritional sciences, public health, humans, Brazil

## Abstract

Background: The lack of a national table informing the caffeine contents in foods, drinks, dietary supplements, and medications sold in Brazil added to the noncompulsory disclosure of caffeine contents on labels of food products makes it difficult to estimate caffeine consumption in the Brazilian population. Therefore, this study aimed to develop the Brazilian Caffeine Content Table (BraCaffT). Methods: A systematic search for caffeine levels in foods, drinks, recipes, supplements, and medications was performed through a literature review, afterward collecting data from the United States Department of Agriculture Food Data Central, information from manufacturers’ and websites, and the Brazilian official medication guide. Subsequently, data systematization was performed in a spreadsheet with standardized values presented in mg of caffeine per 100 g or 100 mL of food or drink and a capsule or pill for medications and some dietary supplements. Results: The BraCaffT presents 57 items, divided into 11 categories: coffees, teas and infusions, cocoa powder, chocolates, cocoa-based beverages, desserts, soft drinks, energy drinks, guaraná powder, dietary supplements, and medications. Conclusions: The BraCaffT emerges as an instrument of great relevance and wide applicability in clinical contexts, in academic research, and as a database for the Brazilian population to better understand the amounts of caffeine in foods, drinks, dietary supplements, and medications consumed daily favoring a safe intake.

## 1. Introduction

Caffeine is found in over 63 plant species, is widely present in the food culture of several countries, and is the most consumed psychoactive substance in the world [1,2]. Several foods and beverages commonly consumed in Brazil contain caffeine, such as coffee, green tea, cocoa, chocolate, and yerba mate derivatives such as chimarrão. Caffeine is also included in energy drinks, dietary supplements, and medications [3,4]. Furthermore, coffee is the main source of caffeine for the Brazilian population, being the most consumed food and nonalcoholic beverage in the country [5,6,7,8].

The primary motives for the consumption of caffeine are due to its ability to promote acute benefits such as the reduction of fatigue, increased wakefulness, improved attention and cognitive performance, analgesia, and augmentation of athletic ability [9,10]. On the other hand, several regulatory agencies worldwide have published guidelines about safe levels of caffeine consumption [11,12,13,14,15,16], and overall, they indicate that high caffeine consumption (>400 mg/day) may promote negative effects on sleep, mood, physical performance, memory, and general health [12,15,17,18]. The risks are potentially high for populations more vulnerable to caffeine, such as pregnant women, children, and individuals with heart disease and/or hypertension [19,20]. Despite these international guidelines, there are no caffeine-specific consumption guidelines established by regulatory agencies in Brazil. Furthermore, many individuals are unaware of the amount of caffeine they habitually consume and its health implications [21,22,23]. In part, this can be explained by the lack of caffeine content information in food composition tables and the absence of the mandatory inclusion of caffeine amounts in product labels in several countries, including Brazil. However, applying the caffeine content database of tables from other countries may present some limitations, as they do not incorporate several foods typically consumed in Brazil, in addition to the high variability in caffeine levels due to distinct environmental conditions of cultivation, processing, and food preparation methods adopted in different regions/countries [22,23,24,25].

Therefore, it is necessary to develop a national caffeine content table to provide data regarding the caffeine contents of foods, drinks, dietary supplements, and medications consumed in Brazil. The development of a national caffeine content table will provide a database for Brazilians to consult the typical amounts of caffeine in foods, drinks, dietary supplements, and medications consumed daily. In addition, the table can be used by nutritionists and physicians in clinical practice and also by researchers to assess caffeine consumption in an academic context. Thus, the present study aimed to develop the Brazilian Caffeine Content Table (BraCaffT), including the caffeine contents of foods, drinks, dietary supplements, and medications commonly consumed in Brazil.

## 2. Materials and Methods

This was a methodological study for the development of the BraCaffT that includes foods, drinks, dietary supplements, and medications commonly consumed in Brazil. The study was performed in three stages: (i) a survey was performed to identify the main caffeine source categories of foods, drinks, dietary supplements, and medications consumed in Brazil; (ii) a systematic search for the caffeine contents in the foods, drinks, dietary supplements, and medications consumed by the Brazilian population determined in the first stage; and (iii) data synthesis and quantification.

### 2.1. Category Development Survey

A survey was performed to identify the main caffeine sources of foods, drinks, dietary supplements, and medications consumed in Brazil. For this purpose, initially, data were extracted from the “Household Budget Survey: Analysis of Personal Food Consumption in Brazil (2017–2018)”, which provides information about the individual food intake of the Brazilian population above 10 years old [8]. Some food categories were also created according to studies regarding the analysis of caffeine contents in foods consumed in Brazil [3,26,27,28] and from a study that determined the main sources of caffeine consumed by Brazilians [7]. Subsequently, international studies that compiled foods, drinks, dietary supplements, and medications with caffeine [29,30,31,32] and the United States Department of Agriculture Food Data Central (USDA FoodData Central) (https://fdc.nal.usda.gov/ accessed on 3 June 2020) were analyzed to include foods, drinks, dietary supplements, and medications that are commercialized on the Brazilian market. Finally, an expert panel consisting of two nutritionists and two caffeine researchers (doctoral degree) reviewed the list to assess the presented categories.

### 2.2. Caffeine Content in Foods, Drinks, Dietary Supplements, and Medications

A systematic review of the literature was carried out, including studies that analyzed the caffeine contents in foods and beverages; in addition, data from the USDA were also collected. The caffeine contents in dietary supplements were obtained according to label data and information provided by manufacturers and sellers. To determine the caffeine contents in medications, the medication guide of the National Health Surveillance Agency was used [33]. Dessert recipes were obtained from widely accessible websites in Brazil and calculated according to food caffeine content data previously provided on BraCaffT for the ingredients of each preparation.

#### Literature Search

The literature search was performed in June of 2022 in PubMed and Scielo databases using the terms “caffeine content” in combination with: “coffee”, “tea”, “cocoa”, “chocolate”, “soft drink”, “guarana”, and “energy drinks”, using “Title and Abstract” as a filter. A more extensive search was applied for yerba mate derivatives (chimarrão and tereré) due to the regional consumption and scarcity of studies in the reviewed databases. Therefore, Google Scholar was reviewed using the following search strategy (caffeine OR cafeína) AND (Ilex paraguariensis OR yerba mate OR erva mate) AND (chimarrão AND tereré), using “Title and Abstract” as the filter.

As inclusion criteria, we selected studies that performed the caffeine content analysis of foods and drinks consumed in Brazil (identified in stage one). Therefore, book chapters, review articles, and those using data from other studies were excluded. In addition, the following exclusion criteria were used: records that were not in English, Portuguese, or Spanish or those that did not present the specific caffeine content information necessary for data synthesis and quantification. After screening the studies by analysis of the title and abstract and removing any duplicates, the articles were read in full to assess the selection criteria. Additionally, we scrutinized the references within the identified papers.

The selected studies were analyzed to collect the caffeine contents of the presented foods and drinks. Studies performed in Brazil were prioritized. When the caffeine contents for products were not available in Brazilian studies, the data were obtained from studies performed in other countries containing foods marketed in Brazil and from the USDA FoodData Central. The caffeine content in dietary supplements was collected through the labels, manufacturers’ website information, and, when not available, on websites selling these products. The caffeine contents in medications were obtained from the medication guide of the Brazilian National Health Surveillance Agency [33].

Regarding prepared desserts containing caffeine, a Google search was performed to identify the most widely visited Brazilian recipe websites, and four were selected: (i) tudogostoso.com.br, (ii) receitas.globo.com, (iii) naminhapanela.com, and (iv) receiteria.com.br. A search for recipes with caffeine-containing ingredients was carried out on these websites, and 12 recipes were selected based upon the highest recurrence, namely: chocolate pudding, chocolate mousse, brownie, chocolate cake, brigadeiro (traditional Brazilian dessert made from condensed milk and butter) with chocolate powder, brigadeiro with cocoa, brigadeiro with milk chocolate, coffee pudding, coffee cake, coffee brigadeiro, coffee mousse, and tiramisu. For each dessert, ingredients from 3 different recipes/websites were entered into Google Sheets to estimate the total weight of the recipes, using yield factors where necessary for weight correction, to determine the caffeine contents of the ready-to-eat preparations. The caffeine contents of the ingredients were based upon the previous data extraction.

**Figure 1:**
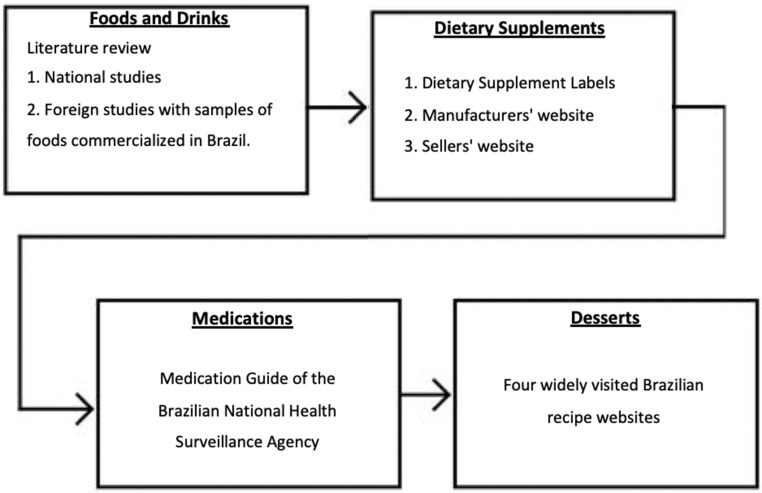
Diagram of databases and priority order to obtain the caffeine contents.

Figure 1 summarizes the search strategy for the caffeine contents information in foods, drinks, dietary supplements, medications, and desserts, including the databases and the priority order.

### 2.3. Data Synthesis and Quantification

After extracting the caffeine contents of foods, drinks, dietary supplements, and medications through the systematic search procedure, all data were added into the Google Sheets platform (Mountain View, CA, USA). First, the caffeine content data were standardized in mg/100 g of food or mg/100 mL of drink and then entered into the spreadsheet, including the references for each item. When the study did not present the caffeine values as mg/100 g or mg/100 mL, the value provided was converted to this standard. For desserts, the caffeine contents were also calculated in mg/100 g of ready-to-eat foods. For powdered dietary supplements, the values provided on the labels, manufacturers’ website, or selling websites were converted to mg/100 g of product to maintain the standardization. Dietary supplements in capsules or pills and medications were standardized in units of capsules or pills.

Following data entry, the foods were grouped according to the following categories: (i) coffees, (ii) teas and infusions, (iii) cocoa powder, (iv) chocolate, (v) cocoa-based beverages, (vi) desserts, (vii) soft drinks, (viii) energy drinks, (ix) guaraná powder, (x) dietary supplements, and (xi) medications. Subsequently, the mean caffeine content was calculated for each item using the different analyzed samples of the same item provided by each study (intra-study mean value). Afterwards, the mean caffeine content between sources (labels, manufacturers’ and websites’ information, medications guides, and USDA FoodData Central) was estimated for each item (between-sources mean value). In addition, the standard deviation (SD); the coefficient of variance (CV); and the minimum and maximum values of each item of food, drink, dietary supplement, and medication were determined. The caffeine contents of items obtained from a single source was described without calculating the SD and CV.

In the coffees category, the caffeine contents for the three varieties of coffee with milk presented in the table were calculated based on the caffeine contents presented in the brewed coffee (Arabica) and the proportion of coffee/milk used in the preparation of each item (80/20%—strong, 50/50%—moderate, and 20/80%—weak (pingado, Brazilian nomenclature).

Some products in the dietary supplements and medications categories showed a standardized value of caffeine contents among the different analyzed brands. In this case, the statistical mode was applied to determine the caffeine value for the category, and, when necessary, the products were separated into subcategories.

Only two food items presented outlier values from the mean caffeine contents of their subcategories and were excluded from the table: (i) a decaffeinated instant coffee with a caffeine content 95 times higher than the average for this item and (ii) a cappuccino with caffeine values 6.4 times lower than the average for the beverage. In addition, two dietary supplements and two medications showed different amounts of caffeine in relation to the statistical mode defined for their category and were also excluded: (i) caffeine anhydrous from growth supplements (Tijucas, Santa Catarina, Brazil) with 420 mg of caffeine per serving contrasting 200 mg of the statistical mode for the category and (ii) caffeine gel from Vitafor (Araçoiaba da Serra, São Paulo, Brazil) with 75 mg of caffeine per serving contrasting 167 mg of the statistical mode and (i) Engov from Hypera Pharma (Itapevi, São Paulo, Brazil) containing 50 mg and (ii) Cefalium from Aché Laboratórios Farmacêuticos (Guarulhos, São Paulo, Brazil) with 75 mg of caffeine per unit contrasting 100 and 65 mg representing the statistical mode for the analgesic category.

The final data were tabulated in order to structure the BraCaffT.

## 3. Results

### 3.1. Literature Search Results

**Figure 2:**
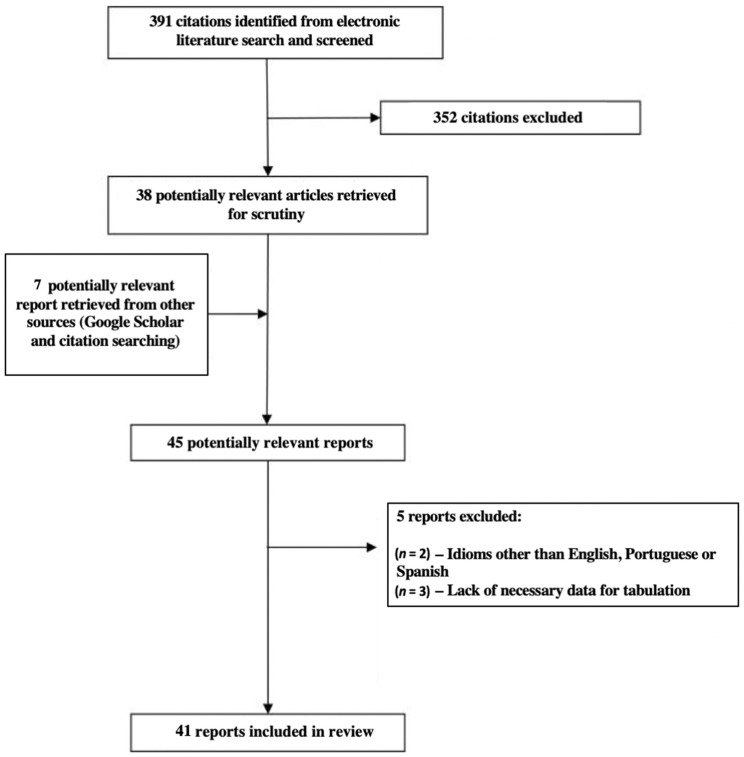
Flowchart of the search and selection of the articles.

From the literature review, 391 articles were identified, of which 352 were excluded due to not meeting the inclusion criteria, resulting in 38 retrieved articles. Seven reports were identified from Google Scholar and via the reference lists of the previously collected articles, reaching a total of 45 screened studies. Of these, five articles were excluded due to no available data on the caffeine contents (*n* = 3), or they were published in other languages (Polish *n* = 1 and Turkish *n* = 1). Finally, 40 articles were selected for the development of the BraCaffT. Figure 2 shows the literature search flowchart.

From the reports included in the literature review, 512 food items were initially collected, of which 6 were considered outliers and excluded from the calculations, as well as 67 that are not present on the Brazilian market or are not included in the Brazilian food culture. Thus, 439 items were used for calculations, of which 149 referred to Brazilian samples. For only two samples of instant coffee, Nescafé Classic (Freehold, NJ, USA) and Nescafé Select Decaffeinated (Freehold, NJ, USA), it was not possible to define the country of origin.

### 3.2. Brazilian Caffeine Content Table (BraCaffT)

The calculated caffeine content data obtained through the systematic search procedure for foods (literature review and USDA FoodData Central); dietary supplements (labels, manufacturers’, and websites’ information); medications (official medications guides); and desserts (Brazilian recipe websites) are shown in Table 1. The Portuguese version of the BraCaffT is presented in Supplementary Table S1.

The BraCaffT presents 57 items (foods, drinks, dietary supplements, and medications) available on the Brazilian market divided into 11 categories with their respective mean caffeine levels (when applied). The coffee category has fourteen subcategories, divided according to their methods of preparation, namely: (i) caffeinated brewed coffee, (ii) espresso, (iii) espresso capsule, (iv) instant coffee (soluble) diluted, (v) decaffeinated brewed coffee, (vi) decaffeinated espresso capsule, (vii) decaffeinated instant coffee (soluble) diluted, (viii) Frappuccino, (ix) cappuccino, (x) brewed coffee with milk (strong: 80% coffee: 20% milk), (xi) brewed coffee with milk (moderate: 50% coffee: 50% milk), and (xii) brewed coffee with milk (pingado: 20% coffee: 80% milk). In addition to these subcategories, three additional ones were created for powdered coffee: (xiii) arabica coffee (Coffea arabica species), (xiv) blend (combinations of several species), and (xv) instant coffee (soluble) powder.

The teas and infusions category was divided into the following subcategories: (i) green, (ii) black, (iii) rooibos (red tea), (iv) mate tea, (v) iced tea, (vi) chimarrão, and (vii) tereré. Furthermore, the chocolate category was divided into three subcategories: (i) milk chocolate, (ii) semisweet, and (iii) dark.

Following, the desserts category is composed of: (i) coffee pudding, (ii) coffee cake, (iii) coffee brigadeiro, (iv) coffee mousse, (v) tiramisu, (vi) chocolate pudding, (vii) chocolate mousse, (viii) brownie, (ix) chocolate cake, (x) brigadeiro with chocolate powder, (xi) brigadeiro with cocoa, and (xii) brigadeiro with chocolate milk. The soft drinks were subdivided into (i) cola-based and (ii) guaraná-based types.

The dietary supplements category includes eight subcategories: (i) anhydrous caffeine, (ii) pre-workout supplements, (iii) caffeinated energy bars, (iv) caffeinated energy gel, and (v) protein supplements with caffeine. The thermogenic subcategory was further divided into three types: (vi) capsules with low caffeine contents (154 mg), (vii) capsules with high caffeine contents (concentrate) (420 mg), and (viii) powders with higher levels of caffeine (900 mg/100 g).

Lastly, medications were categorized into anti-inflammatory, analgesic, and myorelaxant, and the latter two were subdivided according to low (30 mg) or high (65 or 100 mg and 50 mg) caffeine contents.

## 4. Discussion

To the best of our knowledge, BraCaffT is the first caffeine content table that includes regional foods, drinks, dietary supplements, and medications commercialized in Brazil. Moreover, the systematic method applied covered the scientific literature, the USDA FoodData Central, the labels, manufacturers’ and websites’ information, and the Brazilian official medications guides. Studies published regarding caffeine content tables are scarce; therefore, this innovative research may encourage other research groups to develop caffeine content tables specific to their countries.

Despite existing guidelines on safe caffeine intake limits, official tables of caffeine contents in foods around the world are scarce, and most countries do not include this information in their national food composition tables [12,14,15,16]. Furthermore, some studies have compiled caffeine content data presented in foods, drinks, dietary supplements, and medications [30,31,32,35]; however, none of them proposed to develop a specific caffeine content table but only presented the data applied to other aims of the study. Moreover, the distinct climate and environmental conditions and the different food processing methods, in addition to the varied coffee preparations forms and coffee-included food recipes, can result in large variations in the caffeine content for the same food in different countries. Evaluating the caffeine contents in the regional food context can guarantee more reliable data [27]. Therefore, the BraCaffT was developed considering foods and beverages habitually consumed and the dietary supplements and medications commercialized in Brazil, which makes the table reliable and applicable in the personal, clinical, and research contexts for Brazil.

The most referenced table in the world is the USDA National Nutrient Caffeine Database, which presents 43 sources of caffeine commonly consumed in the United States (US). The strength of the USDA caffeine database is the laboratorial analysis performed on the products collected from the US market. However, the caffeine content is presented in household measures of each product/food without a standard by weight/volume (e.g., mg/100 g or ml). In addition, some foods, such as teas, were included in the same category disregarding the caffeine content variability between the different types of teas, e.g., green tea, red tea, and black tea, as were presented by the BraCaffT (20 mg, 16 mg, and 18 mg). This limitation was considered in the BraCaffT due to the differing levels of caffeine observed in different teas and coffees, as well as the standardization of caffeine contents in mg/100 g or ml [36].

Despite some limitations, and due to the absence of national tables, the USDA caffeine database is used by several studies as a source of information on caffeine contents, assuming the limitation that the database does not represent the local food market (for studies outside the US) [32,35,37,38]. In addition, other studies performed their own caffeine analyses that increase the cost and the complexity of the study [39,40,41]. Nonetheless, the BraCaffT was developed using laboratory data from various studies, preferentially performed in Brazil, with foods commonly commercialized in the country. However, when the caffeine content was not available from Brazilian data, values from studies performed outside of Brazil and the USDA caffeine database were included.

In general, the highest amounts of caffeine per 100 g of food are found in powdered versions of coffee, such as instant coffee (3344 mg/100 g), powder coffee, arabica (1165 mg/100 g), and powder coffee, blend (1444 mg/100 g), of which usually only small portions are used for the preparation of beverages. This is observed in the lower caffeine values found in ready-to-drink beverages prepared from these food sources, such as the diluted version of instant coffee (36 mg/100 mL). In addition, the BraCaffT contents are in agreement with other studies from other countries where the average caffeine content found for instant coffees was 3300 mg/100 mL (ranging from 1600 to 4400 mg/100 mL) [42,43,44].

In the coffee category, the highest caffeine content was observed in the prepared espresso coffee (279 mg/100 mL), while the lowest was found in decaffeinated coffee beverages (capsule: 3 mg/100 mL, brewed: 2 mg/100 mL, and instant: 1 mg/100 mL). These findings are in accordance with a study involving products on the Spanish market, where the highest caffeine content was observed in espresso and the lowest in decaffeinated coffee [41]. Therefore, the consumption of three cups of Brazilian espresso (40 mL each) per day will provide a caffeine intake of 336 mg, which remains below the limit of 400 mg/day proposed by the main international regulatory agencies [12,15].

In the category of teas and infusions, the infusions chimarrão and tereré (26 and 24 mg/100 mL) had the highest levels of caffeine. In addition, the items present in this category have lower concentrations of caffeine when compared to soluble and brewed coffee (36 and 30 mg/100 mL). This was also observed in studies that compared caffeine contents between coffee and tea samples [3,40,41].

It must be noted that several items in the coffee, teas, and infusions categories presented high SDs and CVs. This was expected due to the different food species analyzed, cultivation conditions, and the extraction and processing methods applied that can change the caffeine contents in the beverages [24,25,26,27]. This high variability between and within sources was attested by McCusker et al. (2003) in a study that analyzed the caffeine contents of 20 different coffees purchased at coffee shops in the US. A large variation of caffeine contents was seen for brewed coffee (from 76 to 112 mg/240 mL) and for the same type of coffee purchased at the same coffee shop on six different days (from 130 to 282 mg/240 mL) [24]. Despite the large variability, the BraCaffT provides an approximate mean value that can be used to guide decisions on caffeine ingestion, while the minimum and maximum values provide added information regarding the potential caution that should be taken to avoid excessive consumption.

Cocoa powder presented a caffeine content of 230 mg/100 g, and the levels of caffeine found in its derivatives, such as chocolate and cocoa-derived beverages, is due to the caffeine levels found in cocoa [45,46]. The highest caffeine content was observed in dark chocolate (114 mg/100 g), while the lowest was found in cocoa-based beverages (3 mg/100 g) such as chocolate milk and hot chocolate, likely due to the dilution in liquid. In addition, it is important to note that the CV for caffeine content is particularly high for desserts, as can be seen in coffee cake (CV = 110.2%), chocolate mousse (CV = 90.9%), and brownie (CV = 59.3%), which was expected due to the diversity of recipes with different amounts of caffeine sources (coffee and cocoa powder) used for the recipes.

Regarding industrialized beverages, soft drinks had lower amounts of caffeine when compared to energy drinks, which can contain up to three times more caffeine per 100 mL (e.g., 1 and 9 mg/100 mL for guaraná and cola soda vs. 30 mg/100 mL for energy drinks). Due to the similarity in caffeine contents between different energy drinks, the values are provided as a general measure for all energy drinks (mean caffeine content: 30 mg/100 mL). In a study that quantified caffeine in 37 energy drinks sold in Brazil, and whose data were included in the BraCaffT, no energy drink exceeded the stipulated safety limits for caffeine (400 mg/day) according to the portions indicated on the labels [47]. However, the authors warned that consumers of energy drinks should avoid consumption of several doses in short periods of time and emphasized that consumption by children and adolescents is discouraged by health authorities [12,15,47].

Pre-workout powder supplements presented the highest caffeine contents in the dietary supplements category and in the entire BraCaffT (3620 mg/100 g). In addition, they showed the highest caffeine variabilities between samples (SD: 1011 mg/100 g, ranging from 2800 to 4878 mg/100 g). These high levels of caffeine were observed in Da Costa et al. (2022) [48], who analyzed the caffeine contents in pre-workout supplements marketed in Brazil, where five products exceeded the caffeine doses indicated on the labels and the safe intake limit proposed by the international regulatory agencies (400 mg/day) [12,15,48]. According to the International Olympic Committee and the International Society of Sports Nutrition, doses of caffeine between 3 and 6 mg/kg per body weight, consumed approximately 60 min before physical exercise are effective in improving sports performance [49,50]. Caffeine intakes above safe limits (e.g., >400 mg/day) can result in adverse effects, such as insomnia, irritation, nausea, palpitation, agitation, and tachycardia [51,52]. Despite the high concentrations of caffeine in pre-workout supplements, these products usually come with a small 5 g doser and suggest the maximum consumption of two doses per day (totaling 10 g of supplement), which corresponds to a daily consumption of 360 mg of caffeine, the equivalent of three espressos (120 mL = 336 mg). This represents 4.8 mg/kg for a 75 kg individual and would be in accordance with the recommended dose for the ergogenic effects of caffeine in sports performance. However, given the high concentration of caffeine in these products, as stated by Da Costa et al. (2022), pre-workout supplement consumers should reduce their daily intake of other caffeine-containing products, in addition to avoiding taking two or more pre-workout doses on the same day [48]. Furthermore, users of pre-workouts supplements should be widely discouraged from increasing the daily dose of these products due to the increased potential for a caffeine overdose when consumed in powder form [53,54,55].

In the BraCaffT, caffeine supplements in capsules showed a standardized value of 200 mg/capsule. This dosage may be preferable for athletes and individuals who seek more control over the caffeine dose consumed due to the removal of the content variability observed in food sources [50] and pre-workout supplements. This represents 2.7 mg/kg for a 75 kg individual and would be just below the recommended range for those seeking the ergogenic effects of caffeine for sports (3–6 mg/kg), though studies exist showing doses lower than 3 mg/kg to be ergogenic. Another category that presented a standardized caffeine content was medications, which has several products with caffeine ranging from 30 (Myorelaxant B and Analgesic E) to 50 (Myorelaxant A and Anti-inflammatory) and 65 (Analgesic D) to 100 mg/capsule (Analgesic C). The presence of caffeine in analgesic formulations has adjuvant properties increasing the analgesic effect, which justifies its use in medications of this class [56]. With the exception of the levels observed in Analgesic C, similar values have been described for products present on the US market, where pain relief drugs have 30–65 mg of caffeine per dose [20]. The high caffeine values for Analgesic C refer to medications for the treatment of headaches and migraines: Cefaliv (Guarulhos, São Paulo, Brazil), Enxak (Jandira, São Paulo, Brazil), and Migraliv (Hortolândia, São Paulo, Brazil).

Hitherto, there is no caffeine content table developed according to the eating habits of the Brazilian population. Therefore, the BraCaffT emerges as a database of great importance and wide applicability in clinical contexts, for doctors and nutritionists, in academic research for studies analyzing caffeine consumption in the sports science and health context, and by institutes and organizations to assist public health policies. In addition, the BraCaffT will serve as a database for the Brazilian population to better understand the amounts of caffeine present in foods, drinks, dietary supplements, and medications consumed daily, favoring a safe intake and trend to reduce the incidence of adverse effects due to the exaggerated consumption of caffeine.

BraCaffT is the first caffeine content table representative of the foods, drinks, dietary supplements, and medications commercialized in Brazil. Although the table was developed specifically for the Brazilian population, due to the absence of regional studies analyzing the caffeine contents for some included foods, some data were obtained from studies performed in other countries, as well as USDA data. This limitation must be overcome, as new studies of the caffeine content analysis in foods present in the Brazilian market are performed. Moreover, the main limitation of the BraCaffT resides in the high variability of the caffeine contents found in some food and drink sources and dietary supplements. The CV, SD, and minimum and maximum values presented with each item in the table provide important additional information. Therefore, the BraCaffT dataset should be applied with caution due to the impossibility of providing more assertive data, mostly for some categories such as pre-workout and thermogenic supplements, coffee, tea and infusions, and desserts with coffee or cocoa. Since the dietary supplements had their contents calculated according to the labels and information provided by the manufacturers, it is important to emphasize that the declared values may be in disagreement with the actual caffeine content in the product. This nonconformity has already been observed in studies carried out in Brazil that reported significant differences between the caffeine content disclosed on supplement labels and the actual levels of caffeine in products [57,58]. Finally, the BraCaffT can be updated over time according to new published studies on the caffeine contents of Brazilian foods and dietary supplements.

## 5. Conclusions

The Brazilian caffeine content table was developed via a systematic procedure including food and drink, dietary supplements, and medications commonly consumed in Brazil. The BraCaffT presented 57 items divided into 11 categories composed of coffees, teas and infusions, cocoa powder, chocolates, cocoa-based beverages, desserts, soft drinks, energy drinks, guaraná powder, dietary supplements, and medications, with standardized values in mg of caffeine per 100 g or 100 mL and a capsule or pill for medications and some dietary supplements. The BraCaffT emerges as an instrument of great relevance and wide applicability in clinical and academic contexts and for the Brazilian population to better understand the amounts of caffeine in foods, drinks, dietary supplements, and medications consumed daily favoring safe intakes. However, further studies are needed to analyze the caffeine contents of Brazilian foods and products presented in the table to provide more accurate values in the next updated version of the table.

## Figures and Tables

**Table 1:** Brazilian caffeine content table (BraCaffT).

Foods	Caffeine (mg/100 g or mL)	SD (mg)	CV (%)	Min. (mg/100 g or mL)	Max. (mg/100 g or mL)	Serving (mg/per unit)	Common Household Serving ^€^
**Coffees ** ** ^‡^ **							
Brewed Coffee, Arábica	30	13	41	11	54	45	1 coffee cup (150 mL)
Powder Coffee, Arábica	1165	163	14	1050	1280	117	1 tablespoon (10 g)
Powder Coffee, Blend	1444	283	20	1270	1770	144	1 tablespoon (10 g)
Espresso Coffee	279	144	52	177	380	112	1 espresso cup (40 mL)
Capsule Coffee	64	30	47	30	125	64	1 capsule (6 g)
Instant Coffee (soluble), powder	3344	N.A.	N.A.	N.A.	N.A.	67	1 coffee spoon (2 g)
Instant Coffee (soluble), diluted	36	14	39	20	45	54	1 coffee cup (150 mL)
Decaffeinated Brewed Coffee	2	N.A.	N.A.	N.A.	N.A.	3	1 coffee cup (150 mL)
Decaffeinated Capsule Coffee, Nespresso	3	N.A.	N.A.	N.A.	N.A.	N.A.	1 capsule (6 g)
Decaffeinated Instant Coffee	1	1	106	0	2	2	1 coffee cup (150 mL)
Frappuccino Coffee, Starbucks	25	2	9	23	26	88	1 tall cup (350 mL)
Cappuccino Coffee	32	6	18	28	36	48	1 coffee cup (150 mL)
Brewed Coffee, Arábica with milk (80% coffee: 20% milk)	24	N.A.	N.A.	9	43	48	1 cup (200 mL)
Brewed Coffee, Arábica with milk (50% coffee: 50% milk)	15	N.A.	N.A.	5	27	30	1 cup (200 mL)
Brewed Coffee, Arábica with milk—Pingado (20% coffee: 80% milk)	6	N.A.	N.A.	2	11	12	1 cup (200 mL)
**Teas and Infusions ** ** ^‡^ **							
Green Tea, infused	20	2	12	17	21	40	1 tea cup (200 mL)
Black Tea, infused (English breakfast; Earl Grey)	18	5	30	12	32	36	1 tea cup (200 mL)
Mate Tea, infused	5	2	47	3	6	10	1 tea cup (200 mL)
Yerba Mate, Chimarrão	26	15	58	14	52	91	1 chimarrão gourd (350 mL)
Yerba Mate, Tereré	24	12	45	17	36	84	1 tereré gourd (350 mL)
Rooibos Tea (red), infused	16	N.A.	N.A.	N.A.	N.A.	32	1 tea cup (200 mL)
Iced Tea	6	1	18	4	7	18	1 bottle (300 mL)
**Cocoa**							
Cocoa, powder	230	N.A.	N.A.	N.A.	N.A.	23	1 tablespoon (10 g)
**Chocolate**							
Milk chocolate	19	N.A.	N.A.	N.A.	N.A.	9	1/2 bar (45 g)
Semisweet Chocolate	70	N.A.	N.A.	N.A.	N.A.	32	1/2 bar (45 g)
Dark Chocolate	114	N.A.	N.A.	N.A.	N.A.	51	1/2 bar (45 g)
**Cocoa-based beverages**							
Cocoa-based beverages *	3	2	58	2	6	6	1 cup (200 mL)
**Desserts**							
Coffee Pudding	22	8	36	N.A.	N.A.	22	1 dessert cup (100 mL)
Coffee Cake	35	38	110	6	78	21	1 slice (60 g)
Coffee Brigadeiro	39	16	42	28	57	20	1/2 dessert cup (50 mL)
Coffee Mousse	67	27	40	48	98	67	1 dessert cup (100 mL)
Tiramisu	9	4	38	7	13	4	1 slice (45 g)
Chocolate Pudding	10	3	30	7	13	10	1 dessert cup (100 mL)
Chocolate Mousse	13	12	91	6	27	13	1 dessert cup (100 mL)
Brownie	18	10	59	11	29	8	1 piece (45 g)
Chocolate Cake	9	2	29	7	11	5	1 slice (60 g)
Brigadeiro with Chocolate Powder	9	2	22	7	11	5	1/2 dessert cup (50 mL)
Brigadeiro with Cocoa	9	3	35	5	11	5	1/2 dessert cup (50 mL)
Brigadeiro with Chocolate Milk	3	0	4	3	3	2	1/2 dessert cup (50 mL)
**Soft Drinks**							
Guaraná Soda	1	N.A.	N.A.	N.A.	N.A.	4	1 can (350 mL)
Cola Soda	9	1	9	8	10	32	1 can (350 mL)
**Energy Drink**							
Energy Drink **	30	3	10	24	34	75	1 can (250 mL)
**Guaraná**							
Guaraná, powder	3044	1380	45	2068	4020	61	1 coffee spoon (2 g)
**Dietary Supplements**							
Caffeine (anhydrous) ^¥^	200	N.A.	N.A.	N.A.	N.A.	N.A.	N.A.
Energy Bar with Caffeine ***	233	98	42	130	375	82	1 bar (35 g)
Energy Gel with Caffeine ****	167	N.A.	N.A.	N.A.	N.A.	70	1 sachet (30 mL)
Protein Supplement with Caffeine, powder	347	87	25	286	409	104	1 scoop (30 g)
Pre-workout Supplement, powder	3620	1012	28	2800	4878	181	1 teaspoon/doser (5 g)
Thermogenic Supplement, powder	900	196	22	670	1200	45	1 teaspoon/doser (5 g)
Thermogenic Supplement (concentrate) ^¥^	420	N.A.	N.A.	N.A.	N.A.	N.A.	N.A.
Thermogenic Supplement ^¥^	154	33	21	125	200	N.A.	N.A.
**Medications ** **^¥^**							
Anti-inflammatory	50	N.A.	N.A.	N.A.	N.A.	N.A.	N.A.
Myorelaxant A	50	N.A.	N.A.	N.A.	N.A.	N.A.	N.A.
Myorelaxant B	30	N.A.	N.A.	N.A.	N.A.	N.A.	N.A.
Analgesic C	100	N.A.	N.A.	N.A.	N.A.	N.A.	N.A.
Analgesic D	65	N.A.	N.A.	N.A.	N.A.	N.A.	N.A.
Analgesic E	30	N.A.	N.A.	N.A.	N.A.	N.A.	N.A.

SD: standard deviation; CV: coefficient of variation; Min: lowest content observed; Max: highest content observed. ^‡^: The items in the Coffee and the items in the Tea and Infusions categories represent ready-to-drink beverages, with the exception of three items presented in powder form: Powder Coffee, Arabica; Powder Coffee, Blend; and Instant Coffee (soluble), powder. *: Chocolate milk and hot chocolate; **: Red Bull (Fuschl am See, Salzburgo, Austria); Red Bull Sugar Free (Fuschl am See, Salzburgo, Austria); Monster (Weston, Massachusetts, EUA); Monster Sugar Free (Weston, Massachusetts, EUA). ***: Kimera Energy Bar Coffee with Chocolate (Iridium Labs, São Paulo, São Paulo, Brazil); Whey Grego Bar Coffee Cream (Nutrata, Xaxim, Santa Catarina, Brazil); Extreme Bar (GoldNutrition, Lisboa, Lisboa, Portugal); Salted Caramel Energy Bar (Dobro, Moema, São Paulo, Brazil); Cinnamon Maca Energy Bar MINI (Dobro, Moema, São Paulo, Brazil). ****: Go! Energy Gel Caffeine (Athletica Nutrition, Matão, São Paulo, Brazil); VO2 Gel X-Caffeine (Integralmédica, Embu Guaçu, São Paulo, Brazil). ^¥^: Portion: 1 pill, capsule. Myorelaxant and analgesic medications name in Portuguese (Brazil): (A) Dorflex, Miorrelax, Nevralgex, Fenaflex ODC, Dorilax, Benoflex P, Doricin, Ana-flex, Novralflex, Relaflex, and Sedalex. (B) Tandene; Tanderalgin; Miosan caf. C: Cefaliv; Enxak; Migraliv. D: Cefadrin; Sonridor caf; Tylenol DC; Cafiaspirina; Tylalgin CAF; Doril enxaqueca. E: Neosaldina, Benegrip, Coristina D, Doralgina, Melhoral, Sedamed, Doril, Calmador, and Gripinew. ^€^: The household measures for the dietary supplements, soft drinks, and energy drinks were based on the label information, while foods and recipes measures were stipulated in accordance with the GloboDiet food photography manual [34]. N.A.: Not applied.

## Data Availability

Not applicable.

## Supplementary Materials

The following supporting information can be downloaded at: https://www.mdpi.com/article/10.3390/nu14204417/s1: Table S1: Portuguese version of the table—Tabela Brasileira de Teor de Cafeína.
