# Supplementary material for: Development of a Caffeine Content Table for Foods, Drinks, Medications and Supplements Typically Consumed by the Brazilian Population
Source: Nutrients. 2022 Oct 21;14(20):4417. doi: 10.3390/nu14204417 (PMC9608580; doi:10.3390/nu14204417)
Supplement: Supplementary file 1 [file nutrients-14-04417-s001.zip › nutrients-1954124-supplementary.pdf]

nutrients

MDPI

Supplementary Table S1. Tabela Brasileira de Teor de Cafeína (BraCaffT).

Alimentos	Cafeína (mg/100 g ou ml)	DP (mg)	CV (%)	Min. (mg/100g ou ml)	Máx. (mg/100g ou ml)	Porção (mg/ por unidade)	Medida Caseira[€] (unidade)
**Café[‡]**							
Café Coado, Arábica	30	13	41	11	54	45	1 xícara de café (150 ml)
Café em pó, Arábica	1165	163	14	1050	1280	117	1 colher de sopa (10 g)
Café em pó, Blend	1444	283	20	1270	1770	144	1 colher de sopa (10 g)
Café Espresso	279	144	52	177	380	112	1 xícara de espresso (40 ml)
Café em Cápsula	64	30	47	30	125	64	1 cápsula (6 g)
Café Instantâneo (Solúvel), em pó	3344	-	-	-	-	67	1 colher de café (2 g)
Café Instantâneo (Solúvel), diluído	36	14	39	20	45	54	1 xícara de café (150 ml)
Café Descafeinado Coado	2	-	-	-	-	3	1 xícara de café (150 ml)
Café Descafeinado em Cápsula , Nespresso	3	-	-	-	-	-	1 cápsula (6 g)
Café Descafeinado Instantâneo (Solúvel), diluído	1	1	106	0	2	2	1 cápsula (150 ml)
Café do tipo Frappuccino, Starbucks	25	2	9	23	26	88	1 copo grande (350 ml)
Café do tipo Cappuccino	32	6	18	28	36	48	1 xícara de café (150 ml)
Café Coado, Arábica com leite (80% café : 20% leite)	24	-	-	9	43	48	1 xícara (200 ml)
Café Coado , Arábica com leite (50% café : 50% leite)	15	-	-	5	27	30	1 xícara (200 ml)
Café Coado , Arábica com leite – Pingado (20% café : 80% leite)	6	-	-	2	11	12	1 xícara (200 ml)
**Chás e Infusões[‡]**							
Chá Verde, infusão	20	2	12	17	21	40	1 xícara de chá (200 ml)
Chá Preto, infusão (English breakfast; Earl Grey)	18	5	30	12	32	36	1 xícara de chá (200 ml)
Chá Mate, infusão	5	2	47	3	6	10	1 xícara de chá (200 ml)
Chimarrão	26	15	58	14	52	91	1 cuia de chimarrão (350 ml)

Tereré	24	12	45	17	36	84	1 cuia de tereré (350 ml)
Chá vermelho, infusão	16	-	-	-	-	32	1 xícara de chá (200 ml)
Ice Tea (Chá Gelado)	6	1	18	4	7	18	1 garrafa P (300 ml)
**Cacau**							
Cacau, em pó	230	-	-	-	-	23	1 colher de sopa (10 g)
**Chocolate**							
Chocolate ao Leite	19	-	-	-	-	9	1/2 barra (45 g)
Chocolate Meio Amargo	70	-	-	-	-	32	1/2 barra (45 g)
Chocolate Amargo	114	-	-	-	-	51	1/2 barra (45 g)
**Bebidas com Cacau**							
Bebidas com Cacau*	3	2	58	2	6	6	1 xícara (200 ml)
**Sobremesas**							
Pudim de Café	22	8	36	-	-	22	1 taça de sobremesa (100 ml)
Bolo de Café	35	38	110	6	78	21	1 fatia (60 g)
Brigadeiro de Café	39	16	42	28	57	20	1/2 taça de sobremesa (50 ml)
Mousse de Café	67	27	40	48	98	67	1 taça de sobremesa (100 ml)
Tiramisu	9	4	38	7	13	4	1 fatia (45 g)
Pudim de Chocolate	10	3	30	7	13	10	1 taça de sobremesa (100 ml)
Mousse de Chocolate	13	12	91	6	27	13	1 taça de sobremesa (100 ml)
Brownie	18	10	59	11	29	8	1 fatia (45 g)
Bolo de Chocolate	9	2	29	7	11	5	1 fatia (60 g)
Brigadeiro com Chocolate em pó	9	2	22	7	11	5	1/2 taça de sobremesa (50 ml)
Brigadeiro com Cacau	9	3	35	5	11	5	1/2 taça de sobremesa (50 ml)
Brigadeiro com Achocolatado	3	0	4	3	3	2	1/2 taça de sobremesa (50 ml)
**Refrigerante**							
Refrigerante de Guaraná	1	-	-	-	-	4	1 lata (350 ml)
Refrigerante de Cola	9	1	9	8	10	32	1 lata (350 ml)
**Bebida Energética/ Energético**							
Bebida Energética/ Energético**	30	3	10	24	34	75	1 lata (250 ml)
**Guaraná**							
Guaraná, em pó	3044	1380	45	2068	4020	61	1 colher de café (2 g)

**Suplementos Alimentares**	**Cafeína**	**DP (mg)**	**CV (%)**	**Min.**	**Máx.**	**Porção**	**Medida Caseira (unidade)**

	**(mg/100g ou ml)**			**(mg/100g ou ml)**	**(mg/100g ou ml)**	**(mg/ por unidade)**	
Cafeína (anidra)¥	200	-	-	-	-	N.A.	N.A.
Barra Energética com Cafeína ***	233	98	42	130	375	82	1 barra (35 g)
Gel Energético com Cafeína ****	167	-	-	-	-	70	1 sachê (30 ml)
Suplemento Proteico com Cafeína, em pó	347	87	25	286	409	104	1 dosador (30 g)
Suplemento Pré-Treino, em pó	3620	1012	28	2800	4878	181	1 colher de chá/ dosador (5 g)
Suplemento Termogênico, em pó	900	196	22	670	1200	45	1 colher de chá/ dosador (5 g)
Suplemento Termogênico (concentrado)¥	420	-	-	-	-	N.A.	N.A.
Suplemento Termogênico¥	154	33	21	125	200	N.A.	N.A.
**Medicações**¥	**Cafeína (mg/porção)**	**DP (mg)**	**CV (%)**	**Min. (mg/ porção)**	**Máx. (mg/ porção)**	N.A.	N.A.
Anti-inflamatório	50	-	-	-	-	-	-
Miorrelaxante A	50	-	-	-	-	-	-
Miorrelaxante B	30	-	-	-	-	-	-
Analgésico C	100	-	-	-	-	-	-
Analgésico D	65	-	-	-	-	-	-
Analgésico E	30	-	-	-	-	-	-

DP: desvio padrão; CV: coeficiente de variação; Mín: menor teor observado; Máx: maior teor observado. ‡: Os itens da categoria Café e os itens da categoria Chá e Infusões representam bebidas prontas para beber, com exceção de três itens apresentados em pó: Café em pó, Arábica; Café em pó, Blend e Café Instantâneo (solúvel), em pó. *: Chocolate ao leite e chocolate quente; **: Red Bull (Fuschl am See, Salzburgo, Áustria); Red Bull Sugar Free (Fuschl am See, Salzburgo, Áustria); Monster (Weston, Massachusetts, EUA); Monster Sugar Free (Weston, Massachusetts, EUA). ***: Barra Energética Kimera Café com Chocolate (Iridium Labs, São Paulo, São Paulo, Brasil); Barra de Proteína Whey Grego Coffee Cream (Nutrata, Xaxim, Santa Catarina, Brasil); Extreme Bar (GoldNutrition, Lisboa, Lisboa, Portugal); Barra Energética Caramelo Salteado (Dobro, Moema, São Paulo, Brasil); Barra Energética MINI Canela Mooca (Dobro, Moema, São Paulo, Brasil). ****: Go! Energy Gel Caffeine (Athletica Nutrition, Matão, São Paulo, Brasil); VO2 Gel X-Caffeine (Integralmédica, Embu Guaçu, São Paulo, Brasil). ¥: Porção: 1 cápsula, pílula ou drágea. A: Dorflex, Miorrelax, Nevralgex, Fenaflex ODC, Dorilax, Benoflex P, Doricin, Ana-flex, Novralflex, Relaflex, Sedalex. B: Tandene; Tanderalgin; Miosan caf. C: Cefaliv; Enxak; Migraliv. D: Cefadrin; Sonridor caf; Tylenol DC; Cafiaspirina; Tylalgin CAF; Doril enxaqueca. E: Neosaldina, Benegrip, Coristina D, Doralgina, Melhoral, Sedamed, Doril, Calmador, Gripinew. €: As medidas caseiras para suplementos alimentares, refrigerantes e bebidas energéticas foram baseadas nas informações dos rótulos dos produtos, enquanto as medidas de alimentos e receitas foram estipuladas de acordo com o manual fotográfico de alimentos GloboDiet [1]. N.A.: Não aplicável.

References

1. Crispim, S.P.; Fisberg, S.P. *Manual Fotográfico de Quantificação Alimentar*, 1st ed.; Universidade Federal do Paraná: Curitiba, Brazil, 2017; pp. 1–147. ISBN 9788568566084.
